# Dissect Relationships Between Gene Co-expression and Functional Connectivity in Human Brain

**DOI:** 10.3389/fnins.2021.797849

**Published:** 2021-12-09

**Authors:** Xue Zhang, Yingying Xie, Jie Tang, Wen Qin, Feng Liu, Hao Ding, Yuan Ji, Bingbing Yang, Peng Zhang, Wei Li, Zhaoxiang Ye, Chunshui Yu

**Affiliations:** ^1^Tianjin Key Laboratory of Functional Imaging, Department of Radiology, Tianjin Medical University General Hospital, Tianjin, China; ^2^Key Laboratory of Cancer Prevention and Therapy, Department of Radiology, National Clinical Research Center for Cancer, Tianjin’s Clinical Research Center for Cancer, Tianjin Medical University Cancer Institute and Hospital, Tianjin, China; ^3^CAS Center for Excellence in Brain Science and Intelligence Technology, Chinese Academy of Sciences, Shanghai, China

**Keywords:** functional connectivity, gene co-expression, coupling, network, tensor decomposition algorithm, schizophrenia

## Abstract

Although recent evidence indicates an association between gene co-expression and functional connectivity in human brain, specific association patterns remain largely unknown. Here, using neuroimaging-based functional connectivity data of living brains and brain-wide gene expression data of postmortem brains, we performed comprehensive analyses to dissect relationships between gene co-expression and functional connectivity. We identified 125 connectivity-related genes (20 novel genes) enriched for dendrite extension, signaling pathway and schizophrenia, and 179 gene-related functional connections mainly connecting intra-network regions, especially homologous cortical regions. In addition, 51 genes were associated with connectivity in all brain functional networks and enriched for action potential and schizophrenia; in contrast, 51 genes showed network-specific modulatory effects and enriched for ion transportation. These results indicate that functional connectivity is unequally affected by gene expression, and connectivity-related genes with different biological functions are involved in connectivity modulation of different networks.

## Introduction

Functional connectivity calculated from functional magnetic resonance imaging (fMRI) has been widely used to characterize intrinsic low-frequency synchronization of brain activity at rest between anatomically distinct brain regions (Ogawa et al., 1992). Regions and connections are organized into brain functional networks responsible for such distinct functions as vision, audition, motion, attention, memory, and emotion. Different combinations of connectivity impairments are indicative of different neuropsychiatric disorders, which are useful for diagnosing diseases, monitoring clinical courses, and predicting outcomes (Seeley et al., 2009; Vertes and Bullmore, 2015). Despite functional connectivity is found to be heritable (Jansen et al., 2015), the molecular mechanisms supporting functional connectivity remain largely unknown.

Genome-wide association study (GWAS) is a putative method to identify genetic substrates of neuroimaging phenotypes, such as functional connectivity. Using a discovery dataset of 8428 subjects, a GWAS study has identified several genetic loci that are associated with a few functional connectivity phenotypes (Elliott et al., 2018). However, rather large sample size is needed to identify reliable genetic loci in GWAS studies, and most GWAS-identified loci are located in non-coding regions of the genome. Instead, a bulk of studies have used brain-wide gene expression data from the Allen Human Brain Atlas (AHBA) to identify genes associated with functional connectivity by interrogating spatial correlations between gene co-expression and functional connectivity across brain regions.

A pioneer study reveals that brain regions within a functional network showing strong correlations of brain activity at rest also demonstrate highly correlated gene expression (CGE) and that genes with significant association with functional connectivity are enriched for ion channel and synaptic function (Richiardi et al., 2015). Thereafter, several specific associations between gene expression and functional connectivity have been reported. The long-range cortico-cortical functional connectivity is found to be associated with the co-expression of genes uniquely enriched for the supra-granular layers of the cerebral cortex in humans (Krienen et al., 2016). In human brain functional networks, high inter-modular degree and long connection distance are associated with genes enriched for oxidative metabolism and mitochondria, whereas high intra-modular degree and short connection distance are associated with genes enriched for RNA translation and nuclear components (Vertes et al., 2016). The parallel limbic and somato/motor cortico-striatal functional networks are associated with different sets of genes (Anderson et al., 2018), which is also true for the functional connectivity of different visual subregions (Zhang et al., 2021).

Although these studies have advanced our knowledge on the association between functional connectivity and gene expression in the human brain, there are at least three questions need to be further answered. Prior studies have identified genes associated with the averaged functional connectivity phenotypes derived from a group of subjects. It is still unknown that which expression-connectivity associations are consistently present in most individuals. Heritability analysis indicates that genetic and environmental factors influence functional connectivity architecture with different weights (Ge et al., 2017; Teeuw et al., 2019). It is an open question that which kinds of functional connectivity are prone to be affected by genetic factors (e.g., gene expression). Inter-regional gene expression similarity within brain functional networks is much higher than those between networks (Richiardi et al., 2015), suggesting that the distributed brain functional networks may possess dissociable genetic signatures (Richiardi et al., 2015; Anderson et al., 2018; Zhu et al., 2021). However, we barely know which genes contribute generally to functional connectivity architecture of all functional networks, and which genes contribute specifically to a certain functional network. Answering these questions will largely improve our understanding on the molecular mechanisms of functional connectivity.

**FIGURE 1:**
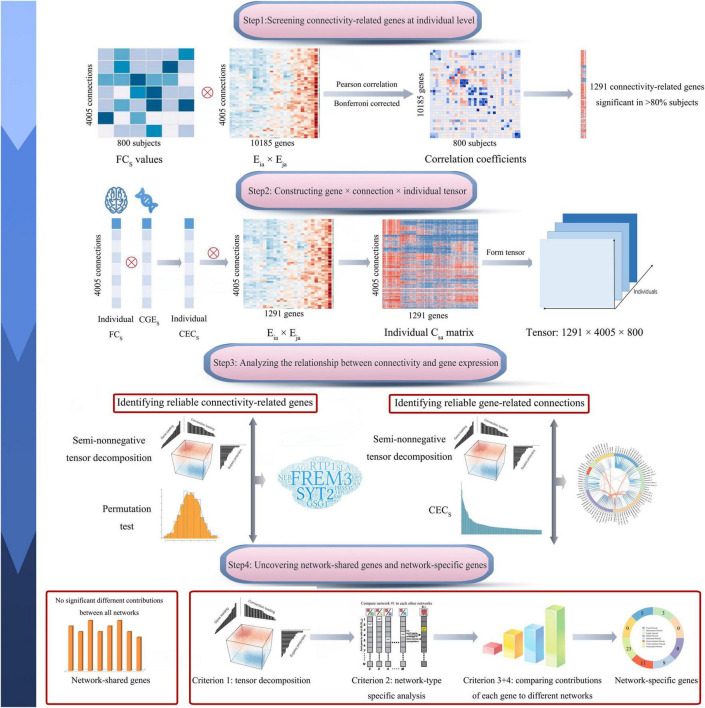
Pipeline of data analysis. In brief, this study includes four steps: screening connectivity-related genes at individual level; constructing gene × connection × individual tensor; identifying connectivity-related genes and gene-related connections; and uncovering network-shared and network-specific connectivity-related genes. The s represents a connection between region i and region j. CEC_s_, connectivity-expression coupling at connection s; CGE_s_, correlated gene expression at connection s; C_sa_, the contribution of gene a to CEC_s_; E_ia_ × E_ja_, the contribution of gene a to the CGE between region i and region j; and FC_s_, normalized functional connectivity strength of connection s.

In this study, we calculated correlations between gene co-expression and functional connectivity across 4005 pairs of brain regions for each of the 800 healthy subjects and identified 1291 genes with significant correlations in most of the 800 subjects (>80%). Then we used multiple comprehensive methods to identify genes associated functional connectivity and functional connectivity more likely affected by gene expression. By assigning 4005 connections into eight functional networks, a series of methods were used to differentiate genes contributing generally to all functional networks and genes contributing specifically to a certain network. The pipeline of this study is shown in Figure 1.

## Materials and Methods

### Calculating Functional Connectivity and Networks

#### Subjects

According to the inclusion criteria of Chinese Han, aged 18–30 years and right handedness and the exclusion criteria of a history of alcohol or drug abuse, a history of neuropsychiatric disorders, and MRI contraindications, we recruited 800 healthy young adults (330 males, 470 females; mean age = 23.8 ± 2.4 years, range: 18–30 years) from the Tianjin Medical University General Hospital (*n* = 400) and Cancer Hospital (*n* = 400). This study was approved by the ethics committee of Tianjin Medical University and all volunteers signed written informed consent before the experiment.

#### MRI Data Acquisition

MRI data from the two hospitals were acquired using the same type of 3.0-Tesla MR scanners (Discovery MR750, General Electric, Milwaukee, WI, United States) with the same scan parameters. The high-resolution structural T1-weighted images were acquired using a brain volume sequence with the following parameters: repetition time (TR) = 8.14 ms; echo time (TE) = 3.17 ms; inversion time (TI) = 450 ms; field of view (FOV) = 256 mm × 256 mm; matrix = 256 × 256; flip angle (FA) = 12°; slice thickness = 1 mm; and 188 sagittal slices. The resting-state fMRI data were obtained using single shot gradient-echo echo-planar imaging (SS-GRE-EPI): TR = 2000 ms; TE = 30 ms; FOV = 220 mm × 220 mm; matrix = 64 × 64; FA = 90°; slice thickness = 3 mm; gap = 1 mm; 40 axial slices; and 180 volumes. During fMRI scans, all subjects were instructed to keep still with their eyes closed, to think of nothing in particular, to stay as motionless as possible, and to not fall asleep.

#### Functional Magnetic Resonance Imaging Data Preprocessing

The resting-state fMRI data were preprocessed using the Statistical Parametric Mapping (SPM12^1^). The first five volumes from each subject were discarded to allow signal to reach equilibrium and ensure the subject to adapt to scanning noise. The acquisition time delay between slices was corrected using sinc-interpolation to make the acquisition time of all voxels consistent within a TR. Head motion of each subject was assessed and corrected using rigid-body transformation. All 800 subjects had acceptable head motion (translational or rotational parameters less than 2 mm or 2°). A unified normalization-segmentation method was used to normalize fMRI images to the Montreal Neurological Institute (MNI) space. fMRI images were coregistered to structural images, and then structural images were segmented and coregistered to the MNI space. The transformation parameters were used to normalize fMRI images to the MNI space. The normalized fMRI images were resampled into 3-mm cubic voxels and smoothed with a Gaussian kernel of 8-mm full-width at half-maximum (FWHM). The frame-wise displacement (FD) was also calculated and time points with FD >0.3 mm were deleted and imputed using cubic spline interpolation. The linear drift, 24 head motion parameters and averaging blood oxygenation level dependent (BOLD) signals of white matter and cerebral spinal fluid were regressed out from the fMRI data. Finally, the fMRI images were filtered with a frequency range of 0.01–0.08 Hz.

#### Constructing Functional Connectivity Matrix and Functional Networks

For each subject, we constructed a functional connectivity matrix (90 × 90) based on the 90 non-cerebellar regions derived from the automatic anatomical labeling (AAL) (Tzourio-Mazoyer et al., 2002) using DPABI (Yan et al., 2016), and then the obtained functional connectivity matrix was used to form a column vector including 4005 independent connections. The final functional connectivity matrix (4005 × 800) was constructed by combining the column vectors of all subjects (*n* = 800). Based on a canonical cortical functional network mask (Yeo et al., 2011), cortical brain regions and their connections were assigned to seven resting-state networks, including the visual network (VN), somatomotor network (SMN), dorsal attention network (DAN), ventral attention network (VAN), fronto-parietal control network (FPN), default-mode network (DMN), and limbic network (LN). And the rest subcortical regions were defined as the subcortical network.

### Gene Expression Data Processing

The normalized microarray gene expression data of two donated brains with the whole brain coverage were obtained from the Allen Institute for Brain Science (AIBS). Gene expression data were processed following a newly proposed pipeline for linking brain-wide gene expression and neuroimaging data (Arnatkevic Iute et al., 2019). Briefly, the latest information from NCBI was used to re-assign probes to genes, and then the noise from gene expression signals was removed. Based on the principle of one probe for one gene, RNA-seq information was used as the reference to select a probe for each gene with more than one probe. Consequently, 10,185 genes were finally selected for 1209 samples according to the pipeline (detailed procedures see Supplementary Material). According to the distance between the coordinate of each sample and the boundary of brain regions in the MNI space, each sample was assigned to a specific region.

### Dissecting Associations Between Gene Expression and Functional Connectivity

#### Screening Connectivity-Related Genes at Individual Level

In each subject, we calculated the CGE score for each pair of brain regions across genes using the following equation:


(1) $$C\,G\,E_{i\,j}=\left(\sum_{a=1}^{N}\left(E_{\text{ia}}\times E_{\text{ja}}\right)\right)/N$$

here, *N* was the total number of genes (*N* = 10,185); i and j represented a pair of brain regions; and E_ia_ and E_ja_ were the normalized expression values (*z*-scores) of gene a in region i and region j. E_ia_ × E_ja_ denoted the contribution of gene a to the global gene co-expression between these two regions. CGE_ij_ was the Pearson correlation coefficient of gene expression between these two regions across all genes, which indicates the similarity of global gene expression between any pair of regions.

In each subject, we could obtain the normalized functional connectivity strength (FC_ij_) and (E_ia_ × E_ja_) for each pair of brain regions. For a given gene (*n* = 10,185) of this subject, we calculated Pearson correlation between FC_ij_ and (E_ia_ × E_ja_) across the 4005 pairs of regions. If the correlation was significant (Bonferroni corrected, *P* < 4.9 × 10^–6^ = 0.05/10,185), this gene was considered to be associated with functional connectivity. These steps were independently conducted in 800 subjects, and only genes with significant correlations with functional connectivity in more than 80% subjects were regarded as connectivity-related genes. The resulting 1291 connectivity-related genes (Supplementary Table 1) were used for the further analyses.

#### Identifying Genes Highly Associated With Functional Connectivity

Two additional methods were used to further identify genes with high and reliable associations with functional connectivity from genes obtained by the individual-level analysis. Before these analyses, we defined the connectivity-expression coupling (CEC_s_) of a connection s (i.e., a pair of brain regions i and j) as the product (FC_s_ × CGE_s_) of the normalized FC_s_ and CGE_s_ of this connection in each subject. The global CEC of this subject was defined as the Pearson correlation coefficient between the normalized FC_s_ and CGE_s_ across the 4005 connections (Eq. 2). *S* was the total number of connections (*S* = 4005 in this study).


(2) $$\text{CEC}=\left(\sum_{s=1}^{S}\left(F\,C_{s}\times C\,G\,E_{s}\right)\right)/S$$

We also calculated the contribution of each gene to the CEC at each connection in each subject using the following Eq. 3:


(3) $$C_{s\,a}=\left(F\,C_{s}\times C\,G\,E_{s}\right)\times \left(E_{i\,a}\times E_{j\,a}\right)$$

here, s represented a pair of brain regions (i and j); FC was the normalized functional connectivity strength; CGE was the normalized correlated gene expression; C_sa_ indicated the contribution of a given gene a to CEC_s_. Based on this equation, a C_sa_ matrix (1291 genes × 4005 connections) was generated for each subject. In the following parts, the population-averaged C_sa_ for each gene and each connection was computed by averaging C_sa_ values of this gene at this connection in the 800 subjects; and the population- and connection-averaged C_sa_ for each gene was computed by averaging C_sa_ values of this gene for all included connections (*n* = 4005 for whole brain connectivity analysis and *n* = the number of connections within a given network for network-level analysis) and subjects (*n* = 800).

##### A Tensor Decomposition Model

The ‘‘*MultiCluster*’’ method^2^ is proposed to explore three-way interactions of genes, tissues, and individuals using semi-nonnegative tensor decomposition (Wang et al., 2019). This approach handles heterogeneity in each dimension and learns the clustering patterns across different dimensions of the data in an unsupervised manner. In this study, we replaced tissue by functional connectivity (*n* = 4005) and replaced gene expression by the C_sa_ of each gene (*n* = 1291). Using the “*MultiCluster*” method, we can identify genes closely associated with functional connectivity and connections more influenced by these genes.

The 400 subjects from each of the two hospitals were randomly divided into two groups, and finally creating four independent groups. The semi-nonnegative tensor decomposition model was used to investigate complex interactions of 1291 genes, 4005 functional connections and 200 individuals of each group (Supplementary Figure 1). This method decomposed tensor into 10 components that represent major data variations in the group. Only the first component was selected for further analyses because this component had much greater output score than other components (Supplementary Table 2 and Supplementary Figure 2). Detail methods for component selection and consistency assessment between groups are described in Supplementary Methods. The component included three vectors of individual, gene and connection. From each vector, we can extract a weight score for each item to represent the relative contribution of the item to the component. We defined genes with high associations with functional connectivity as those with absolute weight scores > (mean + SD) of the absolute weight scores of the 1291 genes.

##### A Permutation Test

The labels of genes and connections were randomly shuffled 1000 times to generate a random distribution of the population- and connection-averaged C_sa_ values of each gene. The significance of each gene was inferred by observing if the true C_sa_ value of this gene was greater than all permutation-derived C_sa_ values of this gene (*P* < 0.001). To further reduce false positive of the identified connectivity-related genes, only genes identified by both tensor decomposition and permutation test were finally considered as genes with high and reliable associations with functional connectivity.

#### Identifying Functional Connectivity Highly Associated With Gene Expression

The functional connections with absolute population-averaged CEC_s_ values greater than the (mean + SD) of all the 4005 connections were defined as connections associated with gene expression. The identified gene-related functional connections were further validated using the tensor decomposition model. From the first component of the tensor decomposition model, we can extract a weight score for each connection from the connection vector to represent the relative contribution of this connection to the component. We defined connections with high associations with gene expression as those with absolute weight scores greater than the (mean + SD) of the 4005 connections.

#### Dissecting Connectivity-Related Genes at the Network Level

The whole brain was divided into eight functional networks, and then functional connections within each functional network were extracted to identify connectivity-related genes common to all functional networks or specific to a certain network.

##### Identifying Genes Shared by Brain Functional Networks

For each gene, one-way analysis of variance (ANOVA) was used to compare the population-averaged C_sa_ values among the eight groups of intra-network connections from different functional networks. Genes without significant difference (*P* ≥ 0.05) across the eight groups were defined as connectivity-related genes common to all functional networks.

##### Identifying Network-Specific Genes

We used conserved criteria to identify network-specific genes. A gene was considered to be specific to a given functional network if the gene satisfied the following four criteria.

###### Identifying Network-Specific Genes by Tensor Decomposition Model

A prerequisite for a network-specific gene is that this gene should be highly correlated with functional connections of the network. The non-negative tensor decomposition algorithm was applied to functional connections of each functional network to identify genes with higher contribution to connections of the functional network. For each network, the connectivity-related genes were defined as those with absolute weight scores > (mean + SD) of all the 1291 genes. The resulting 764 genes were used to further network-specific analyses.

###### Network-Type Specific Analysis

As commonly used in cell-type specific analysis (Dougherty et al., 2010; Xu et al., 2014), the specificity index (SI) was adapted to assess the specificity of a gene to a particular functional network relative to all other networks. Here, cells were replaced by brain functional networks, and gene expression values were replaced by the population-averaged C_sa_ values of each gene for connections within a functional network. For each gene, a *P*-value for SI was calculated *via* the permutation testing (1000 permutations). This method was applied to each gene identified by the network-based tensor decomposition model, and significant genes (*n* = 144) (*P* < 0.001) were used for further network-specific analyses.

###### Comparing Contributions of Genes to Different Networks

For each of the identified candidate genes (*n* = 144), ANOVA was performed to compared the difference in the population-averaged C_sa_ values among the eight groups of intra-network connections from different functional networks. Genes (*n* = 144) with significant difference (Bonferroni corrected, *P* < 3.47 × 10^–4^ = 0.05/144) among the eight groups were selected for further *post hoc* analysis. According to the population- and connection-averaged C_sa_ value of each gene of each network, we can identify the first two functional networks with the greatest contribution from this gene. Two strategies were then used to assess the specificity of this gene to the first functional network. First, the gene was considered to be specific to the first network if its population- and connection-averaged C_sa_ of the first network was at least twice greater than that of the second network. To further assess the significance, a two sample *t*-test was conducted to compare the population-averaged C_sa_ differences (*P* < 0.05) between the two networks. Only genes satisfied the two criteria (twice greater and significant) were considered as genes specific to the first functional network.

### Gene Enrichment Analysis

With regards to functions of our main gene clusters, Toppogene (Chen et al., 2009) was used in gene enrichment analysis,^3^ which calculates the functional similarity to training gene list to prioritize candidate genes. Moreover, associations between connectivity-related genes and common brain disorders were identified by MAGMA (de Leeuw et al., 2015), which provides gene-set analysis based on GWAS data. Among the common neuropsychiatric disorders, autistic spectrum disorder (ASD), attention-deficit/hyperactivity disorder (ADHD), bipolar disorder (BP), major depression disorder (MDD), and schizophrenia (SCZ) were included in our analyses. The GWAS summary statistic results of the five neuropsychiatric disorders were collected from previous studies (Schizophrenia Working Group of the Psychiatric Genomics Consortium, 2014; Autism Spectrum Disorders Working Group of The Psychiatric Genomics Consortium, 2017; Wray et al., 2018; Demontis et al., 2019; Stahl et al., 2019; Supplementary Table 3). For all enrichment analyses, multiple comparisons were corrected d by the Benjamini and Hochberg method of false discovery rate (FDR-BH correction, *P* < 0.05).

## Results

### Genes Associated With Connectivity in Most Individuals

**FIGURE 2:**
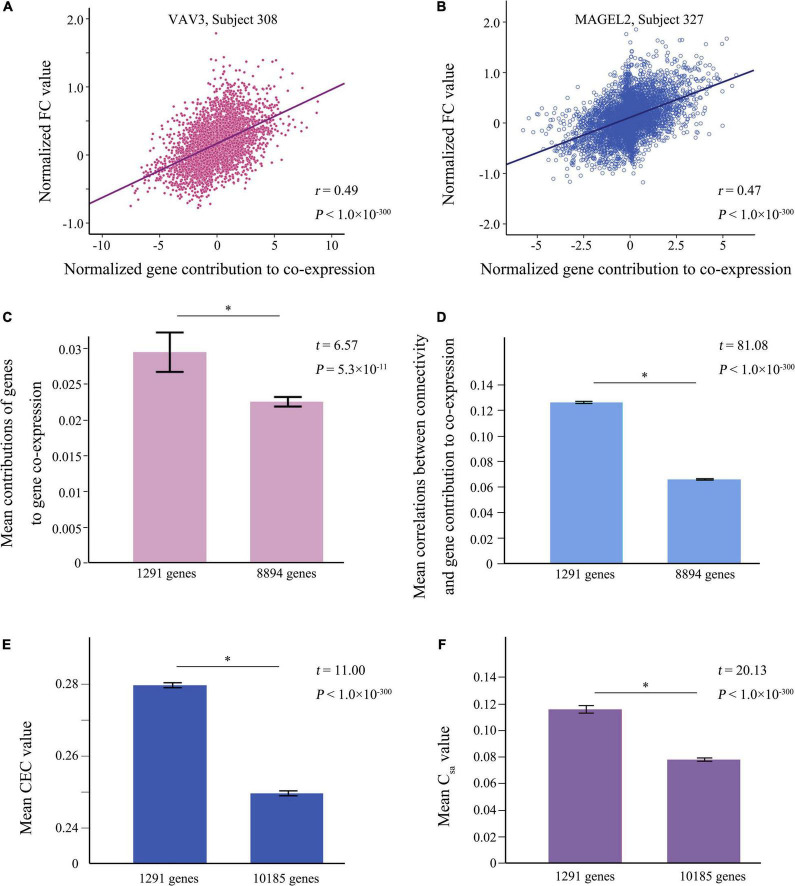
The analysis of the connectivity-related genes. **(A,B)** Are representative correlation maps between functional connectivity and gene contribution to co-expression of *VAV3* in the 308th subject and *MAGEL2* in the 327th subject. **(C)** Shows the mean contributions of 1291 genes to gene co-expression greater (*t* = 6.57, *P* = 5.3 × 10^–11^) than those of the rest 8894 genes. **(D)** Demonstrates the population-averaged correlations between connectivity and the contribution of 1291 genes to gene co-expression stronger (*t* = 81.08 and *P* < 10^–300^) than those of the rest 8894 genes. **(E)** Shows the global CEC values calculated based on the 1291 genes greater (*t* = 11.00, *P* < 10^–300^) than those derived from the 10,185 genes. **(F)** Demonstrates the population- and connection-averaged C_sa_ values of the 1291 genes stronger (*t* = 20.13; *P* < 10^–300^) than those of the 10,185 genes. Mean + SEM for all graphs. The significant difference between two groups was showed as *. CEC, connectivity-expression coupling; C_sa_, the contribution of each gene (a) to the CEC at each connection (s); FC, functional connectivity.

In each subject, connectivity-related genes were identified by detecting significant correlations between FC_ij_ and (E_ia_ × E_ja_) of each gene across the 4005 pairs of brain regions (Bonferroni corrected *P* < 4.9 × 10^–6^ = 0.05/10,185). Correlation maps between FC_ij_ and (E_ia_ × E_ja_) of two representative genes (*VAV3* and *MAGEL2*) in two individuals are shown in Figures 2A,B. By calculating the ratio of a gene present in the significant gene list in the 800 subjects, 1291 connectivity-related genes were identified in at least 80% of these subjects (Supplementary Table 1).

Two sample *t*-test demonstrated that the mean contributions of the 1291 genes were greater (*t* = 6.57, *P* = 5.3 × 10^–11^) than those of the rest 8894 genes (Figure 2C) and the 1291 genes had much stronger population-averaged correlations (*t* = 81.08, *P* < 10^–300^) than the rest 8894 genes (Figure 2D and Supplementary Figure 3).

Moreover, in the 800 subjects, two sample *t*-test demonstrated that the global CEC values calculated based on the 1291 genes were greater (*t* = 11.00, *P* < 10^–300^) than those derived from the 10,185 genes (Figure 2E) and the population- and connection-averaged C_sa_ values of the 1291 genes were also much stronger (*t* = 20.13; *P* < 10^–300^) than those of the 10,185 genes (Figure 2F).

### Genes Highly and Reliably Associated With Functional Connectivity

#### Selecting Connectivity-Related Genes With Tensor Decomposition Model

**FIGURE 3:**
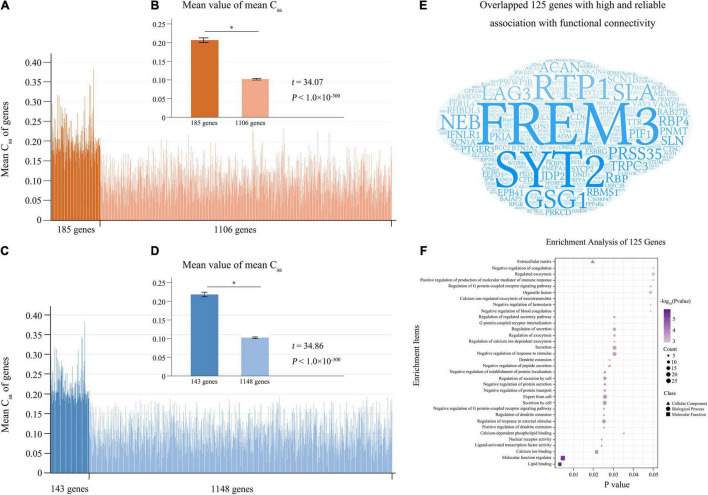
Reliable connectivity-related genes. **(A)** Is the population- and connection-averaged C_sa_ values of the 1291 genes, and the 185 connectivity-related genes identified by tensor decomposition model are marked in dark orange. **(B)** Shows that the 185 genes demonstrate stronger population- and connection-averaged C_sa_ values (*t* = 34.07; *P* < 10^–300^) than the rest 1106 genes. **(C)** Shows the population- and connection-averaged C_sa_ values of the 1291 genes, and the 143 connectivity-related genes identified by the permutation test are marked in dark blue. **(D)** Shows the population- and connection-averaged C_sa_ values of the 143 genes greater (*t* = 34.86; *P* < 10^–300^) than those of the rest 1148 genes. **(E)** Is word-cloud representation of the 125 reliable connectivity-related genes identified by both methods. **(F)** Shows enrichments of the 125 reliable connectivity-related genes. Mean + SEM for all graphs. The significant difference between two groups was showed as *. C_sa_, the contribution of each gene (a) to the connectivity-expression coupling at each connection (s).

From the 10 components derived from the tensor decomposition of each group (*n* = 200), the first component with the largest weight was selected for further analyses (Supplementary Table 2 and Supplementary Figure 2), and this component showed low mismatch rate (0.077) and high component correspondence (mean correlation = 0.998) among the four groups. For each group, we defined connectivity-related genes as those with absolute weight scores > (mean + SD) of the scores of the 1291 genes. With the criterion of the 100% repeated rate among the four groups, we selected 185 candidate connectivity-related genes. In the 1291 genes, two sample *t*-test demonstrated that the population- and connection-averaged C_sa_ values of the 185 genes were much greater (*t* = 34.07; *P* < 10^–300^) than those of the rest 1106 genes (Figures 3A,B).

#### Selecting Connectivity-Related Genes With Permutation Test

A permutation test showed that the population- and connection-averaged C_sa_ values of 143 genes were significantly greater than all permutation-derived C_sa_ values of this gene (*P* < 0.001) (Supplementary Table 4). Two sample *t*-test demonstrated that the population- and connection-averaged C_sa_ values of the 143 genes were much greater (*t* = 34.86; *P* < 10^–300^) than those of the rest 1148 genes (Figures 3C,D).

#### Genes With High and Reliable Association With Functional Connectivity

The 125 genes identified by both tensor decomposition (*n* = 185) and permutation test (*n* = 143) were considered as genes with high and reliable association with functional connectivity (Supplementary Table 4 and Figure 3E). Among the 125 connectivity-related genes, 105 genes have been previously reported as connectivity-related genes (Richiardi et al., 2015; Krienen et al., 2016; Anderson et al., 2018) and 20 genes were novel (Supplementary Table 4). The 125 genes were mainly enriched for the regulation of dendrite extension, response to external stimulus, and G protein-coupled receptor signaling pathway, protein secretion and transport, calcium ion binding (FDR-BH corrected, *P* < 0.05) (Supplementary Table 5 and Figure 3F). Moreover, these genes showed significant association with schizophrenia (FDR-BH corrected, *P* = 0.017).

### Functional Connections Highly Associated With Gene Expression

**FIGURE 4:**
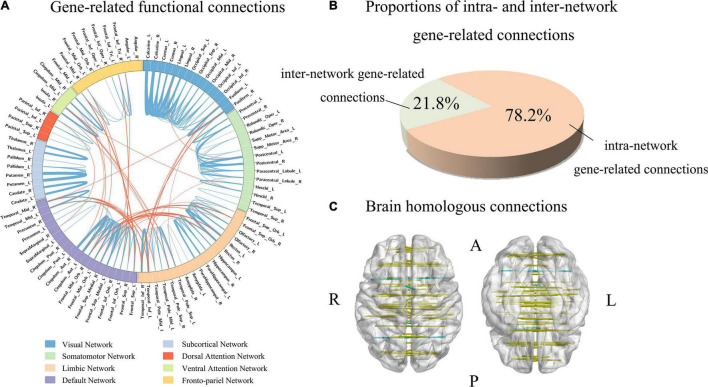
Functional connections associated with gene expression. **(A)** Shows gene-related functional connections with a circle map. The eight functional networks are represented by different colors. The blue lines represent the intra-network functional connections, and the orange lines denote the inter-network functional connections. The thickness of a line indicates the mean strength of the connectivity-expression coupling at each connection. **(B)** Demonstrates the proportions of intra- and inter-network connections in all gene-related connections (*n* = 179). **(C)** Shows that 91% homologous connections are gene-related connections.

Firstly, the absolute value of the population-averaged CEC_s_ score was used to identify functional connections associated with gene expression with a threshold of greater than the (mean + SD) of all the 4005 connections. This method generated 255 gene-related functional connections (Supplementary Table 6 and Supplementary Figure 4). Then we used the tensor decomposition model to independently identify gene-related connections. From the first component of the model, gene-related connections were defined as those with absolute weight scores greater than the (mean + SD) of the 4005 connections, resulting in 180 gene-related connections. Among those connections, the 179 gene-related connections identified by tensor decomposition model were completely included in the 255 gene-related connections identified based on the mean CEC_s_ score. Therefore, the 179 connections were considered as functional connections highly associated with gene expression (Figure 4A and Supplementary Table 6).

In the 179 connections, 140 connections (78.2%) were intra-network connections and 39 (21.8%) were inter-network connections (Figure 4B). In the total of 45 homologous connections between the two hemispheres, 41 homologous connections (91.1%) were identified as gene-related connections (Figure 4C). The 140 intra-network connections were assigned to the eight functional networks, and the number and the percentage of gene-related connections in each network are listed in Table 1.

**TABLE 1:** Intra-network functional connections associated with gene expression.

Functional networks	Numbers of intra-network connections	Numbers of gene-related connections	A (%)	B (%)
Visual network	91	44	31.4	48.4
Somatomotor network	91	23	16.4	25.3
Dorsal attention network	6	3	2.1	50.0
Ventral attention network	6	2	1.4	33.3
Limbic network	153	25	17.9	16.3
Frontoparietal network	45	8	5.7	17.8
Default mode network	153	29	20.7	19.0
Subcortical network	28	6	4.3	21.4
Sum	573	140	100	24.4

*A (%) refers to the ratio of the number (44) of gene-related connections in a given network (such as the visual network) to the total number (140) of gene-related connections in all networks; B (%) refers to the ratio of the number (44) of gene-related connections in a given network (such as the visual network) to the total number (91) of connections in the network.*

### Connectivity-Related Genes Shared by Brain Functional Networks

**FIGURE 5:**
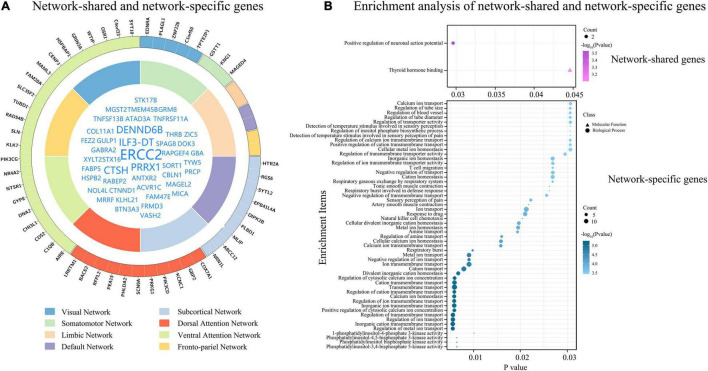
Network-shared and network-specific genes. **(A)** Shows network-shared genes (inner circle) and network-specific genes (outer circle). The eight functional networks are represented by different colors. **(B)** Demonstrates the results of enrichment analyses of the 51 network-shared genes and the 51 network-specific genes.

In the 1291 genes, 51 genes without significant difference (*P* ≥ 0.05) in the population-averaged C_sa_ values among the eight groups were considered as connectivity-related genes common to all functional networks (Figure 5A). These genes were enriched for the positive regulation of neuronal action potential (Supplementary Table 5 and Figure 5B). Moreover, these network-shared connectivity-related genes were also enriched for schizophrenia (FDR-BH corrected, *P* = 0.017).

### Network-Specific Genes

The tensor decomposition model was applied to each functional network to identify 764 genes with higher C_sa_ with the threshold of absolute weight scores > (mean + SD) of all the 1291 genes. The SI was then used to assess the specificity of each of the 764 genes to each network relative to other networks. We found that 144 genes were significantly enriched for a certain functional network (*P* < 0.001). All the 144 genes showed significant difference (Bonferroni corrected, *P* < 3.47 × 10^–4^ = 0.05/144) among the eight groups by ANOVA. To further identify genes specific to each functional network, we assessed the contributions of each gene (*n* = 144) to its first two most associated functional networks with two criteria. Only 51 genes satisfied the two criteria (twice greater and significant) were considered as genes specific to the first functional network (Table 2, Figure 5A, and Supplementary Figure 5). These network-specific genes were mainly enriched for the regulation of ion transport and transmembrane transport, and ion homeostasis (Supplementary Table 5 and Figure 5B).

**TABLE 2:** Numbers of network-specific connectivity-related genes identified by different combinations of criteria.

Functional networks	Criterion 1 only	Criteria 1 + 2	Criteria 1 + 2 + 3 + 4
Visual network	198	7	5
Somatomotor network	106	41	3
Dorsal attention network	150	37	11
Ventral attention network	119	28	23
Limbic network	157	2	0
Frontoparietal network	173	27	0
Default mode network	176	4	0
Subcortical network	133	13	9

*Criterion 1: the gene should have higher C_sa_ in the tensor decomposition model of the functional network; Criterion 2: the gene should show significant enrichment for the functional network in network-type specific analysis; Criterion 3: the population-averaged C_sa_ values of the functional network were significantly greater than those of any other networks; and Criterion 4: the population- and connection-averaged C_sa_ value of the functional network was twice greater than those of any other networks.*

## Discussion

In this study, we performed a comprehensive analysis on associations between functional connectivity and gene co-expression in the human brain. We identified 125 connectivity-related genes (20 novel genes), which are linked to dendrite extension and signaling pathway. Moreover, we identified 179 gene-related connections that are influenced more by gene expression than other connections. Most of gene-related connections were intra-network connections, especially homologous connections. Finally, we identified 51 network-shared genes and 51 network-specific genes, which were involved in different molecular processes (action potential for the former and ion transportation for the latter). These findings may improve our understanding of the molecular mechanisms of functional connectivity in the human brain.

In previously conducted transcription-neuroimaging association studies (Fornito et al., 2011; Oh et al., 2015; Richiardi et al., 2015; Krienen et al., 2016; Vertes et al., 2016; Anderson et al., 2018; Zhang et al., 2021; Zhu et al., 2021), spatial correlations are performed between gene expression and group-averaged neuroimaging maps or inter-group difference maps, which neglect inter-individual variations in neuroimaging measures. In this study, individual variations of functional connections were considered with two strategies: (1) connectivity-expression correlations were conducted at an individual level and only genes with significant correlations in most individuals (>80%) were considered as connectivity-related genes; and (2) a tensor decomposition algorithm was used to simultaneously consider interactions among genes, connections and individuals. To further control false positive results, a permutation test was used to test the significance of each gene derived from both strategies in the connectivity-expression associations. The resulting 125 genes were defined as reliable connectivity-related genes and the correctness and reliability of this finding are supported by the fact that 105 out of 125 (84%) genes have been reported in previous connectivity-expression association studies (Richiardi et al., 2015; Krienen et al., 2016; Anderson et al., 2018). More importantly, 20 novel connectivity-related genes were identified in this study, which may provide new insight or evidence on the molecular mechanisms of functional connectivity.

In consistent with prior studies linking connectivity-related genes to signal transmission processes (Richiardi et al., 2015; Anderson et al., 2018), the identified 125 connectivity-related genes in this study were also enriched for various biological processes associated with signal transmission. Six connectivity-related genes are directly related to G protein-coupled receptor signaling pathway, and many other genes involve in signal transmission by regulating protein secretion and transport, and dendrite extension. Fourteen genes including two novel ones (*MCUB* and *DOC2B*) are associated with Ca^2+^ binding and Ca^2+^-mediated biological processes. As an important second messenger, these Ca^2+^-related biological processes are critical for signal transmission (Bando et al., 2016; Toth et al., 2016). Twenty-five genes including four novel ones (*RIPOR2*, *ADTRP*, *IFNLR1*, and *PMEPA1*) are related to the regulation of response to stimulus, including the immune response. These findings indicate that a series of complex biological processes are involved in the formation, development, and plasticity of functional connectivity.

The identified 125 reliable connectivity-related genes and the 51 network-shared connectivity-related genes were significantly enriched for schizophrenia (34/125 reliable genes and 17/51 network-shared genes) rather than other common psychiatric disorders (ASD, ADHD, BP, and MDD), which is well consistent with the notion that the functional disconnection is the most prominent neuroimaging feature in schizophrenia (van den Heuvel and Fornito, 2014; Dong et al., 2018). These findings indicate that connectivity-related genes identified in healthy subjects may be also related to functional disconnection in schizophrenia. The resulting 51 (34 + 17) connectivity- and schizophrenia-related genes are the potential candidates for investigating the molecular mechanisms underlying the functional disconnection in schizophrenia.

It is well known that functional connectivity is influenced by both genetic and environmental factors (Ge et al., 2017; Teeuw et al., 2019). However, we barely know which kinds of functional connections are prone to be regulated by gene expression. In this study, we identified 179 functional connections that were highly associated with gene expression. Most of the gene-related functional connections (78.2%) were located in the same functional network, which is consistent with the higher correlations between gene expression and functional connectivity within functional networks than between networks (Richiardi et al., 2015; Zhu et al., 2021). Notably, 41/45 (91.1%) homologous connections were identified as gene-related connections, indicating that homologous connections are prone to be regulated by gene expression. This result is also consistent with the knowledge that homologous regions between the bilateral hemispheres have both higher genetic correlations and stronger anatomical connections (Stark et al., 2008; Eyler et al., 2014; Shen et al., 2015; Elliott et al., 2018).

In this study, we identified 51 network-shared genes associated with functional connectivity, which were enriched for positive regulation of neuronal action potential, which is the core biological process in brain activity throughout the brain. Specifically, *CTNND1* is related to adhesion between cells and signal transduction, and is involved in the regulation of protein kinase and signaling receptor binding, WNT signaling pathway, and postsynaptic membrane neurotransmitter receptor levels (Tang et al., 2016). *GABRA2* plays a role in the regulation of GABA-gated chloride ion channel activity and chemical synaptic transmission (Lengeling et al., 1999).

We also identified 51 network-specific connectivity-related genes, which were mainly enriched for the regulation of ion transport and ion homeostasis. Several network-specific genes are involved in various signaling pathways, such as *EDNRA* and *KNG1* are related to the G protein-coupled receptor signaling pathway (Horstmeyer et al., 1996; Sato et al., 2008), *HTR2A* is involved in the CREB and ELK-SRF/GATA4 signaling pathways, and *NR4A2* is associated with canonical WNT signaling pathway (Zagani et al., 2009). Several network-specific genes (*COX7A1*, *SLN, GBP2, BACE2, PRKG1, SYTL2, ABCC12*, and *RGS6*) are related to energy metabolism, such as ATP synthesis and GTPase activity. Several network-specific genes (*PIK3CD, AIRE, PEA15, C1QB*, and *CHI3L1*) play a role in immune response.

Two limitations should be mentioned when one interprets the results of this study. First, brain imaging data and gene expression data were obtained from different subjects and these two groups of subjects differ in age and race. Thus, the spatial correlation analyses between gene co-expression and functional connectivity may be confounded by inter-individual conservation of brain gene expression and inter-group differences in these demographic data. Second, we still do not know if gene–gene spatial autocorrelation is a meaningful biological phenomenon or a meaningless confounding factor, and thus we did not correct for gene–gene spatial autocorrelation in this study, which may bias our findings.

In conclusion, this study provides new knowledge for the relationship between gene expression and functional connectivity in the human brain. Firstly, we confirmed that most of the previously identified connectivity-related genes can be detected in individual-level transcription-neuroimaging association analysis. Secondly, we found unequal influences of gene expression on functional connections and identified 179 functional connections linking more closely to gene expression than other connections. Thirdly, we identified network-shared genes and network-specific genes for the first time, which are involved in different molecular processes. These findings may improve our understanding of the relationship between gene expression and functional connectivity.

## Data Availability Statement

The datasets presented in this study can be found in online repositories. The names of the repository/repositories and accession number(s) can be found in the article/Supplementary Material.

## Ethics Statement

The studies involving human participants were reviewed and approved by the Ethics Committee of Tianjin Medical University. The patients/participants provided their written informed consent to participate in this study. Written informed consent was obtained from the individual(s) for the publication of any potentially identifiable images or data included in this article.

## Author Contributions

WQ: methodology and conceptualization. FL: software and formal analysis. HD, YJ, and BY: visualization. PZ, WL, and ZY: resources. CY: conceptualization, methodology, writing – review and editing, and supervision. All authors contributed to the article and approved the submitted version.

## Conflict of Interest

The authors declare that the research was conducted in the absence of any commercial or financial relationships that could be construed as a potential conflict of interest.

## Publisher’s Note

All claims expressed in this article are solely those of the authors and do not necessarily represent those of their affiliated organizations, or those of the publisher, the editors and the reviewers. Any product that may be evaluated in this article, or claim that may be made by its manufacturer, is not guaranteed or endorsed by the publisher.

## Footnotes

1
http://www.fil.ion.ucl.ac.uk/spm

2
https://github.com/Miaoyanwang/MultiCluster

3
https://toppgene.cchmc.org/
