# Supplementary material for: Dissect Relationships Between Gene Co-expression and Functional Connectivity in Human Brain
Source: Front Neurosci. 2021 Dec 9;15:797849. doi: 10.3389/fnins.2021.797849 (PMC8696273; doi:10.3389/fnins.2021.797849)
Supplement: Supplementary file 1 [file Data_Sheet_1.pdf]

Supplementary Information for

Dissect relationships between gene co-expression and functional connectivity in human brain

Supplementary Methods

1. Parameter selections in processing the human brain-wide AHBA gene expression data

Briefly, the latest information from NCBI was used to re-assign probes to genes, and then the noise from gene expression signals was removed. Based on the principle of one probe for one gene, RNA-seq information was used as the reference to select a probe for each gene with more than one probe. Finally, 10185 genes were selected for 1209 samples according to this pipeline. Each sample was assigned to a specific brain region according to its coordinate in the MNI space. Related parameter selections in processing gene expression data in our study were as follow:

(1) options.ExcludeCBandBS = true;

(2) options.useCUSTprobes = true;

(3) options.updateProbes = 'reannotator' (functions to read new files into matlab has been updated);

(4) options.probeSelections = 'RNAseq';

(5) options.parcellations = 'aparcaseg';

(6) options.distanceThreshold = 2;

(7) options.signalThreshold = 0.5;

(8) options.divideSamples = 'listCortex';

(9) options.excludeHippocampus = false;

(10) options.VARfilter = false;

(11) options.VARscale = 'normal';

(12) options.VARperc = 50;

(13) options.RNAseqThreshold = 0.2;

(14) options.RNAsignThreshold = alse;

(15) options.correctDistance = false;

(16) options.calculateDS = true;

(17) options.distanceCorrection = 'Euclidean';

(18) options.Fit = 'exp';

(19) options.normaliseWhat = 'wholeBrain';

(20) options.normMethod = 'scaledRobustSigmoid'

(21) options.percentDS = 100;

(22) options.saveOutput = true;

(23) options.normaliseWithinSample = true;

(24) options.meanSamples = 'meanSamples'.

2. The principle for selecting candidate components

From the tensor decomposition model, we obtained an output value for each component, which represents the degree of this component in explaining for the whole connectivity-expression coupling tensor. The first component with the greatest output value was firstly selected. The other components will be selected only if their output values were greater than a half of the output value of the first component. Based on the output values of all components (Table S2 and Figure.S2), only the first component was finally selected for further analysis.

5. Assessing component consistency between groups

For the selected first component, we further assessed component consistency by calculating mismatch rate and component correspondence among the four groups (200 subjects for each group). The mismatch rate was calculated based on (S-1) and represented the mean deviation of the identified connectivity-related genes in each group from the gene set identified in all the four

groups. From the tensor decomposition model, we obtained weight scores of all the 1291 genes in the gene vector from each group, and then we calculated Pearson correlations between weight scores derived from every two groups. Their mean correlation coefficient was used to assess component correspondence.

$$\text{Mismatch rate} = (\sum_{g=1}^{4} Num_g - M))/(4\times M) \qquad \text{[S-1]}$$

M is the number of overlapped connectivity-related genes among the 4 groups, and Num_g is the number of connectivity-related genes identified in group g.

Supplementary Figure

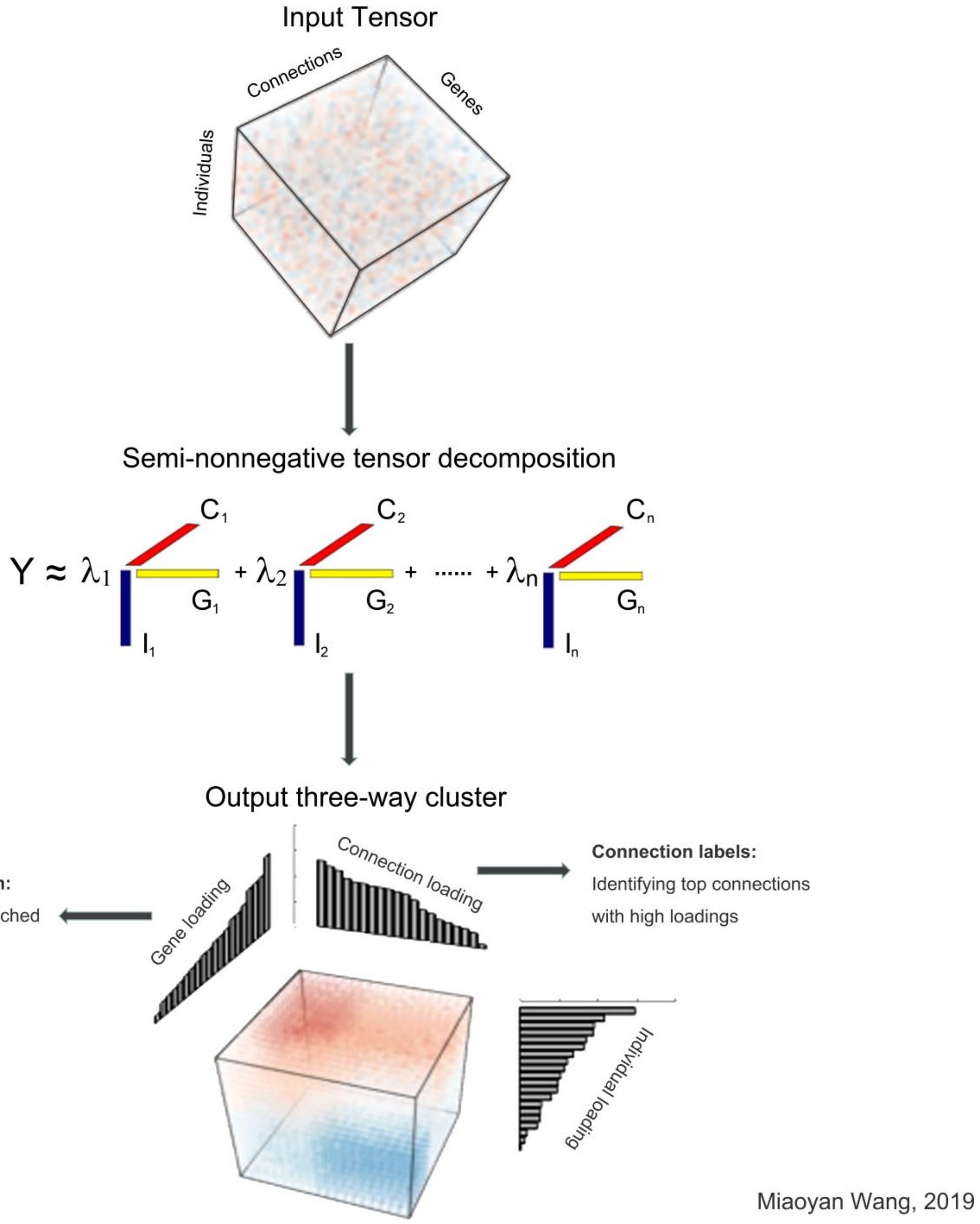

Supplementary Figure 1. Schematic diagram of "*MultiCluster*" method.

The "*MultiCluster*" method based on the semi-nonnegative tensor decomposition processes data follows this schematic diagram provided by Miaoyan. (Miaoyan Wang et al, 2019)

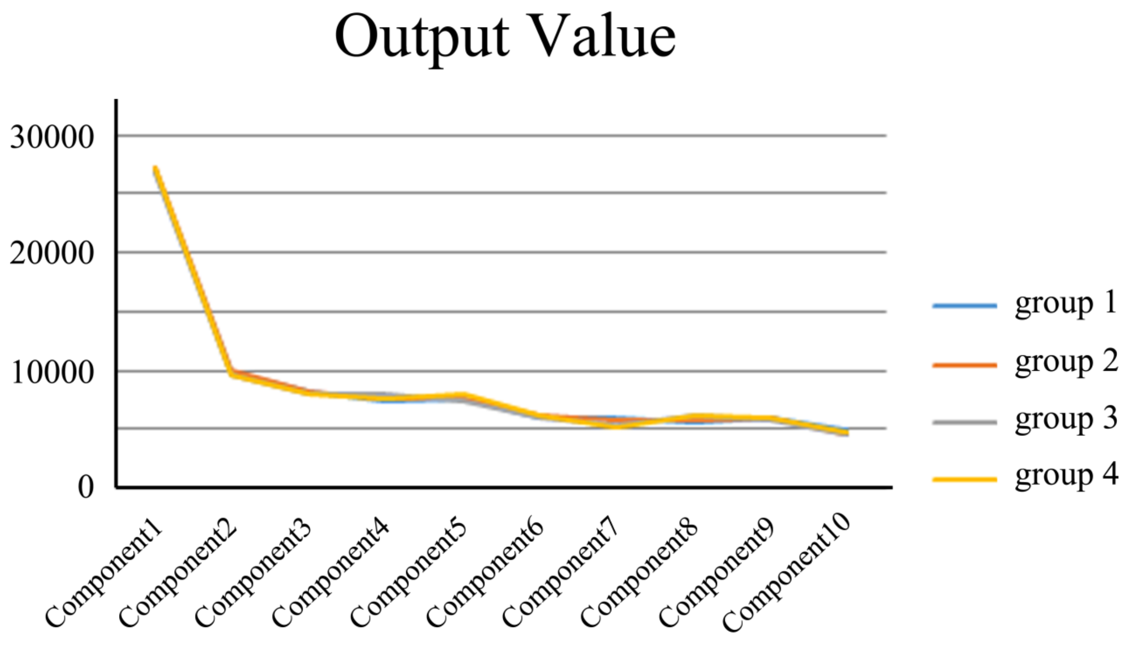

Supplementary Figure 2. The output values of components of four groups from the tensor decomposition.

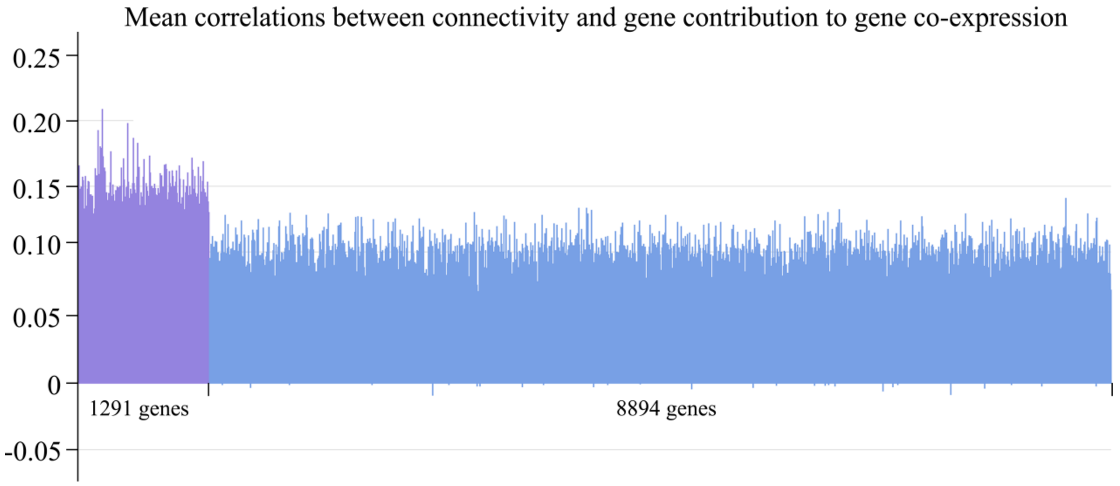

Supplementary Figure 3. The mean correlations between connectivity and gene contribution of 10185 genes to gene co-expression across 800subjects

The mean correlations of functional connectivity and gene contribution of 1291 genes and the rest 8894 genes to gene co-expression across 800 subjects. The mean correlations of 1291 connectivity-related genes are marked in purple.

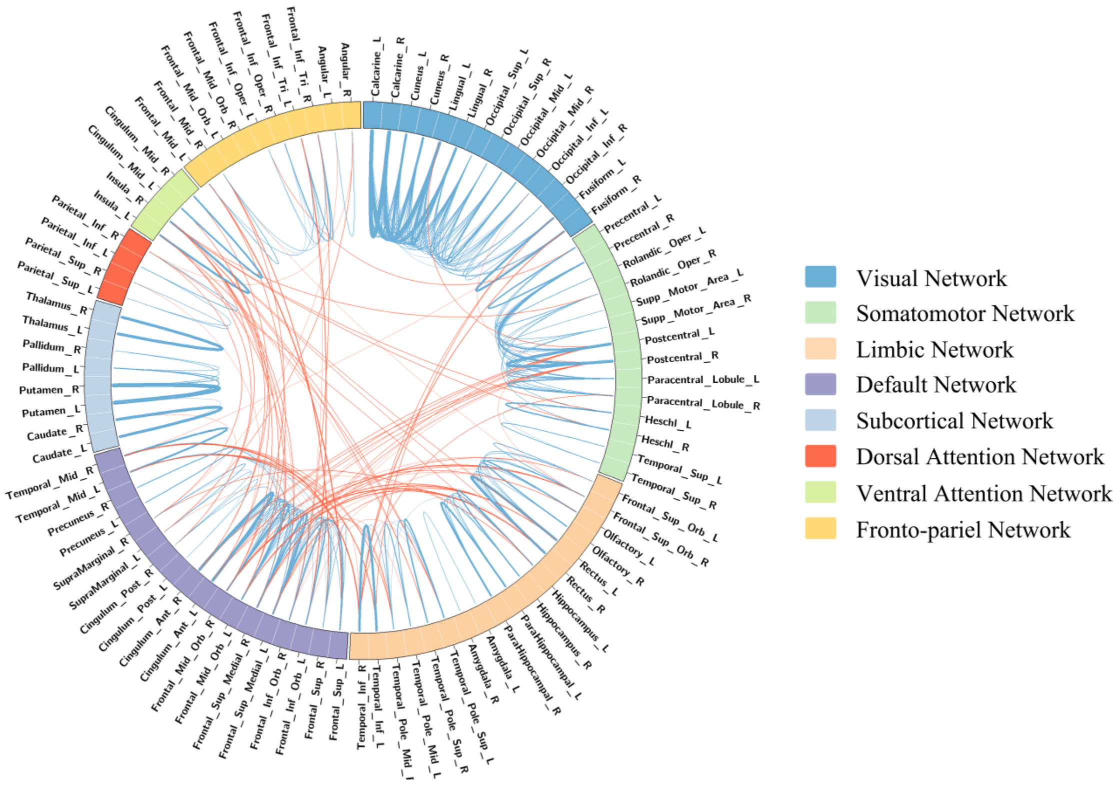

Supplementary Figure 4. 255 functional connections highly associated with gene expression

The circle map shows 255 gene-related functional connections. The 8 functional networks are represented by different colors. The blue lines represent the intra-network functional connections, and the orange lines denote the inter-network functional connections. The thickness of a line indicates the mean strength of connectivity-expression coupling at each connection.

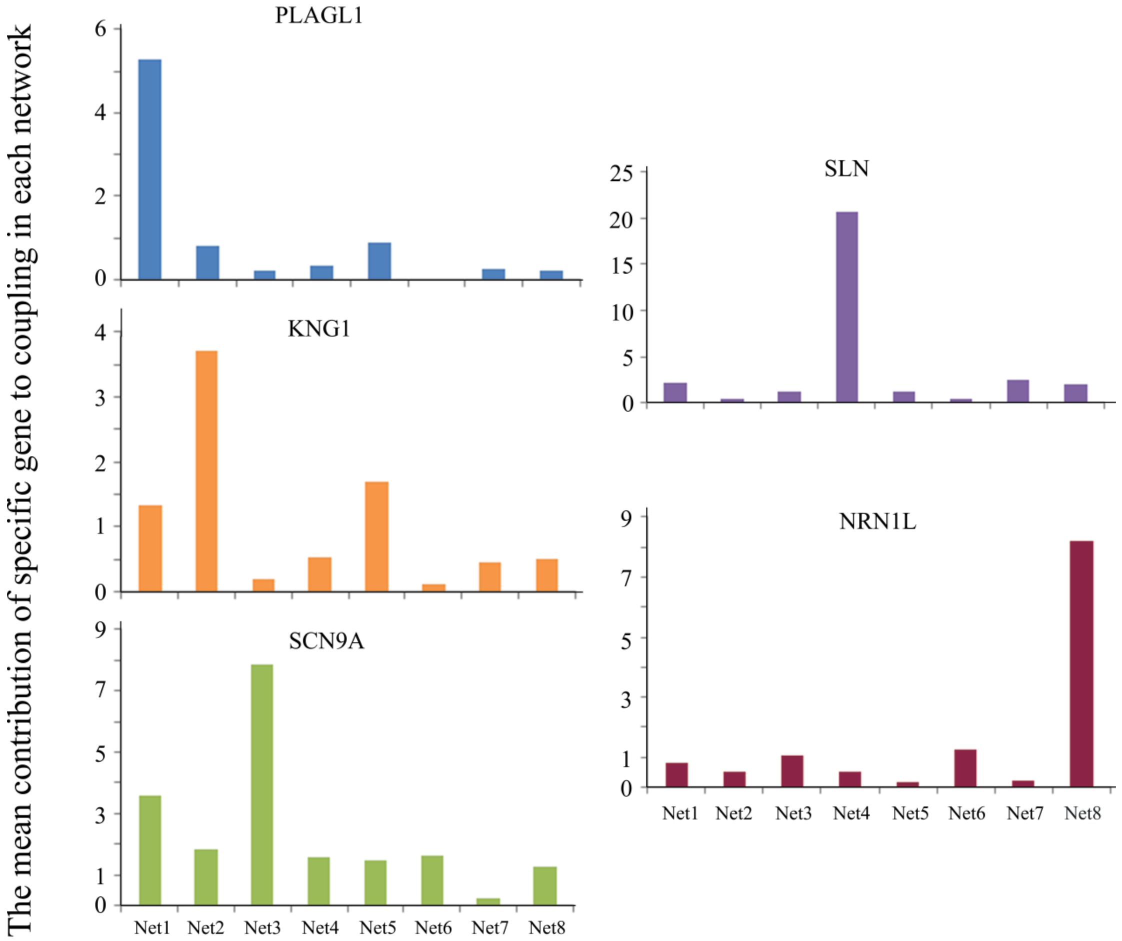

Supplementary Figure 5. The mean contributions of reprehensive network-specific gene to connectivity-expression coupling among all networks

The histogram shows the mean contributions of some reprehensive network-specific genes to connectivity-expression coupling among all networks.

Supplementary Table

Supplementary Table 1. The significant connectivity-related gene number under different threshold.

The ratio of significant connectivity-related genes in 800 subjects	Significant connectivity-related genes number	Significant and reliable connectivity-related genes number with permutation test
100%	0	0
95%	125	12
90%	489	41
85%	852	85
80%	1291	143
75%	1807	199
70%	2361	268
65%	2939	326
60%	3495	396
55%	4035	456
50%	4573	522

We present the main results of genes with significant correlations with functional connectivity in more than 80% subjects in the main context based on conservative estimates.

Supplementary Table 2. The output values of components from tensor decomposition model among four groups.

Output value	Group1	Group2	Group3	Group4
Component 1	27035.5	27204.8	26886.7	27119.2
Component 2	9789.1	9944.1	9558.9	9686.2
Component 3	8107.4	8199.2	8067.6	7995.0
Component 4	7342.4	7637.6	7905.7	7601.2
Component 5	7671.2	7511.2	7478.3	7898.0
Component 6	6045.2	6151.2	6073.7	6125.4
Component 7	5962.5	5769.0	5426.4	5167.0
Component 8	5617.1	5861.1	6234.3	6156.5
Component 9	6032.4	6034.2	5689.5	5882.5
Component 10	4894.8	4657.1	4541.8	4727.0

Supplementary Table 3. Neuropsychiatric disorders data.
Supplementary Table 4. Important gene lists.
Supplementary Table 5. Results of enrichment analyses.
Supplementary Table 6. Important Connections related to gene expression.
